# How can learning effects be measured in Balint groups? Validation of a Balint group questionnaire in China

**DOI:** 10.1186/s12909-021-03030-x

**Published:** 2021-12-09

**Authors:** Kurt Fritzsche, Lili Shi, Johanna Löhlein, Jing Wei, Yue Sha, Yongbiao Xie, Yanling He, Volker Tschuschke, Guido Flatten, Yibo Wang, Chen Jin, Rainer Leonhart

**Affiliations:** 1 Center for Mental Health, Department of Psychosomatic Medicine and Psychotherapy, Medical Center - University of Freiburg, Faculty of Medicine, Hauptstr. 8, D 79104 Freiburg im Breisgau, Germany; 2 Department of Psychological Medicine, Peking Union Medical College Hospital, Chinese Academy of Medical Sciences & Peking Union Medical College, Beijing, China; 3 Guangdong General Hospital, Guangzhou, China; 4 Shanghai Mental Health Center, Shanghai, China; 5 Department of Medical Psychology, University Hospital, University of Cologne, Cologne, Germany; 6 Shanghai Ninth People’s Hospital, Huangpu Branch Hospital, Shanghai, China; 7 Department of Psychiatry, Zhongguancun Hospital, Beijing, China; 8 Institute of Psychology, University of Freiburg, Freiburg, Germany

**Keywords:** Balint group, questionnaire, validation, learning effects, group process, China

## Abstract

**Background:**

Balint groups aim to reflect doctor-patient relationships on the basis of personal cases. This study reports the validation of a questionnaire aimed at the identification of learning processes among Balint group participants in China.

**Methods:**

This multicenter cross-sectional study was conducted during Balint group sessions in Beijing, Guangzhou and Shanghai. A heterogeneous sample of different professional groups was intended to adequately capture the reality of Balint work in China. After a Balint group session, the participants were asked to complete the Mandarin version of the Balint group session questionnaire (BGQ-C) and the group questionnaire (GQ), an internationally validated instrument to assess central dimensions of therapeutic relationships during group processes.

**Results:**

Questionnaires from n = 806 participants from 55 Chinese Balint groups, predominantly comprising individuals with a medical background, were analyzed. Most participants were female (74.6%), and the average age was 34.2 years old (SD = 9.4). The results indicated good to very good reliability (Cronbach’s α = .70 to .86; retest r_s_ = .430 to .697). The verification of the construct validity of the BGQ-C showed satisfying convergent (r_s_ = .465 to .574) and discriminant validity (r_s_ = -.117 to -.209). The model was tested with a confirmatory factor analysis of a three-factor model (standardized root mean square residual = .025; comparative fit index = .977; Tucker-Lewis index = .971). The 3 empirically identified scales resulted in good model fit with the theoretical dimensions of Balint work postulated in the literature: “reflection of transference dynamics in the doctor-patient relationship”, “emotional and cognitive learning” and “case mirroring in the dynamic of the group”. Due to the high correlations between the factors, a single-factor model was possible. A group comparison between the German and Chinese samples showed different loadings across cultures.

**Conclusions:**

The BGQ-C is a quick-to-complete, item-based measuring instrument that allows the relevant dimensions of Balint group work to be recorded. This study suggests good psychometric properties of the Chinese version. Nevertheless, it must be assumed that the composition of constructs in the two countries is different.

## Background

Participation in Balint groups has been a component of medical and psychotherapeutic training in many countries for over 50 years. In accordance with the methodology developed by Michael Balint, doctors present their own case vignettes in a moderated group process with the aim of better understanding aspects of the doctor-patient relationship and improving it in terms of a better treatment relationship [1–3].

Based on psychoanalytic theory, Balint adopted the concept of free association from the dyadic treatment relationship and expanded it by focusing on the doctor-patient relationship within a group method. As a psychiatrist and psychoanalyst, Balint’s concern was initially to make the findings of psychoanalysis useful for somatic medicine as well. Second, his aim was to train doctors to learn how to use their personality and emotions as a tool in treating their patients [4].

Research in relation to the work of Balint groups have thus far been based on very different outcome parameters and have therefore produced very mixed results [5]. Positive effects include improvements in the capacity to empathize [6, 7], changes in conversational behavior that combines a willingness to listen more when talking to patients and reducing their own share of the dialog [8], improvements in psychotherapeutic skills and self-confidence, fewer brooding thoughts about patients, greater work satisfaction, indications of an improved doctor-patient relationship, and significantly reduced burnout [3, 9–13]. However, the methodological quality of the studies is limited; these studies sometimes involve very small sample sizes and are exclusively retrospective surveys of participants [5]. No findings are available for process research regarding the work of Balint groups.

The current German Balint group questionnaire (BGQ-G) makes it possible for the first time to examine which process variables enable a favorable course and a positive effect of Balint work.

### Balint work in China

In the course of the last ten years, the quality of the doctor-patient relationship in China has steadily deteriorated. Patients and doctors greatly mistrust each other. Violent attacks by disgruntled patients against doctors and hospital staff are now routine events. Hospitals in China and the medical profession are regarded as life-threatening. Medical students are reluctant to become doctors [14–16]. The misunderstandings and the mistrust between doctors and patients have social, cultural and economic backgrounds.

Balint group work was already part of the EU project *Postgraduate training in psychosocial medicine for medical doctors in China, Vietnam and Laos* [17]. Between 2005 and 2008, several hundred Chinese doctors participated in this training. This training program largely corresponds to basic psychosomatic care in Germany and includes attendance at Balint groups. It soon became apparent that Chinese doctors greatly appreciated Balint group work. During the Asia-Link program and even after it, they began to conduct their own Balint groups in their hospitals [18]. Research on Balint group work in China is just beginning [19]. One study showed that the use of a Balint group may have contributed to improving the emotional labor and job burnout of nurses in cardiology to a large degree [20]. In another study, Balint groups were shown to be an efficacious, feasible, standardized method of preventing resident burnout in China [21]. In a more recent study from China during the COVID-19 pandemic, it was shown that short-term Balint group activity improved the communication ability and self-efficacy level of front-line nurses to some extent [22].

The research questions in this study focused on the validation and reliability of the German Balint group questionnaire (BGQ-G) in China.

- Hypothesis 1: The reliability of the Chinese questionnaire meets the standard.
- Hypothesis 2: The factor structure found in German-speaking countries can be transferred to China.

In addition to these hypotheses, a comparison of the Chinese data with a German-speaking sample was conducted. If the meaning of the constructs was comparable, this should also be confirmed by a multigroup comparison.

## Methods

### Study design and setting

This multicenter cross-sectional study was conducted between March 2018 and June 2019 during Balint group sessions in Beijing, Guangzhou and Shanghai (located in North, East, and South China).

### Participants

A heterogeneous sample of different professional groups was intended to adequately capture the reality of Balint work in China with this questionnaire. To align with the reality of Balint group practice in China, nonmedical participants in Balint groups such as nursing staff were also included in the survey. The group leaders were specifically asked to select any session of their groups, at the end of which the BGQ-C and the Mandarin version of the Group Questionnaire (GQ-C) should be completed. The instructions to the Balint group leaders included standardized information for the participants, such as voluntary participation and data protection requirements in relation to anonymized surveys.

### Variables and measurement

The research questions related to the validation of the BGQ in China. An analysis was carried out at the item level (i.e., scaling, use of all expressions of the scale), and confirmation of the factor structure of the German-language instrument was assessed.

Following a Balint group session, the participants were asked to fill out the Mandarin version of BGQ-G [23] and the GQ [24], an internationally validated instrument to assess group therapy. Furthermore, sociodemographic data of the participants, professional specialization, information about their previous experiences with Balint work, whether the participants presented their own case and whether participation was mandatory or voluntary were collected. Group leaders completed a questionnaire about age, sex and professional experience in leading Balint groups.

### Development of the German Balint group questionnaire (BGQ-G)

Based on the theory of Balint group work and previous research findings, the following theoretical dimensions of Balint work have been developed [4, 6, 23, 24]:

- Learning experience of medical participants with regard to the doctor-patient relationship
- Diagnostics of the doctor-patient relationship (transfer dynamics)
- Reflection of the presented patient case in the group processes
- Awareness of one’s own proportionate contributions to the doctor-patient relationship
- Significance of group leader interventions

An item pool of 50 questions was developed. The chosen questions seemed appropriate for operational mapping of the theoretical assumptions about the work of Balint groups from the perspective of Balint group participants. After eliminating unsuitable items, a final questionnaire was produced with 17 items. Three factors explained a satisfactory variance of the questions.

All the items with factor loads ≥ 0.65 were very good on only one scale. The reliabilities of the individual scales (based on Cronbach’s alpha) were good to very good for scales 1-3 (between 0.82 and 0.71), although the reliability of scale 4 (0.63) was admittedly in the doubtful range.

The final version of the BGQ-G was developed in two pilot studies (N=91 and N=294) and validated on a large sample of 1,635 participants. Using exploratory and confirmatory factor analyses (structural equation models), a good to very good model fit (CFI = 0.97, RMSEA = 0.054, SRMR = 0.033) was confirmed [23]. The dimensional structure of the BG-Q includes three scales that are independent of each other: (1) reflection of the transference dynamics in the doctor-patient relationship, (2) emotional and cognitive learning, and (3) case mirroring in group dynamics. The three scales represent four out of five dimensions derived from the theory of Balint work.

A total of 12 items could be assigned to the three following scales.

- Scale 1: Reflection of transference dynamics in the doctor-patient relationship (items 2, 10, 13, 15, and 16);
- Scale 2: Emotional and cognitive learning (items 5, 6, 9, and 11); and
- Scale 3: Case mirroring in the dynamic of the group (items 4, 7, and 12).

The reliabilities of the three scales (based on Cronbach’s alpha) were good to very good (between 0.71 and 0.82). The correlations between the scales were between r = .53 and r = .78. The items were recorded on a 6-level rating scale with values from 0 ("does not apply") to 5 ("applies completely"). Items 3, 4, 8 and 12 refer to the processes within the group, while the other items refer to individual processes. Individual scales 1 and 2 and group scale 3 were formed from the items. The individual items were developed on the basis of theory and discussed in several Balint groups. Thus, content validity is assumed.

The development of the BGQ-G took place during a two-year process in cooperation with the German Balint Society (DBG) with participants from Germany, Austria and Switzerland. The aim was to develop a short, non–time-consuming questionnaire that could be used both in clinical practice and in Balint group research, which records relevant dimensions of Balint group work and is capable of reproducing learning and change processes in future studies with repetitive measurements using operationalized parameters.

### The group questionnaire (GQ-G)

In the world of group psychotherapy, there has been a lack of a practicable tool that enables measurement of central group processes. This gap was closed with the development of the group questionnaire (GQ) [25].

The development process took place in two stages. First, a team of experts (experienced group researchers and clinicians) adapted and reduced the set of 80 items that were used in Johnson’s 2005 study [26]. For the present study, the items were created using empirical data and clinical criteria while taking into account the three relationship constructs (positive bonding, positive working and negative relationship). In stage two, the GQ was tested and revised using confirmatory factor analyses.

The GQ-G consists of 30 items, which were answered on a 7-point Likert scale (1: “is not applicable at all” to 7: “is very applicable”). With its three main scales, the GQ-G reflects the central dimensions of therapeutic relationships. The “solidarity” scale measures the extent of cohesion, commitment and empathy in the group. The “work relationship” scale reflects how well the therapist, the surveyed group member and other group members agree in relation to commonly approved tasks and goals. The “negative relationship” scale reflects the extent of conflicts and lack of empathy within the group. The reliability calculated via Cronbach’s α for the solidarity scale was α=0.92. For the working relationship scale, reliability was recorded as α=0.89, and for the negative relationship scale, reliability was α=0.79. The internal consistencies of the subscales were in a range between α=0.60 and α=0.90. The validity information for the individual scales is described in detail by Bormann (2010) [27].

The Chinese version translated for this study showed internal consistencies of α=0.96, α=0.93 and α=0.91 for the three scales and a range between α=0.78 and α=0.93 for the subscales.

### Bias

Possible bias could arise from a larger number of nonrespondents and a trend toward a socially desirable response. This was prevented by the group leaders distributing the questionnaires after the end of the session and then immediately collecting the questionnaires upon completion. The participants were instructed by the group leader to fill out the questionnaires as honestly as possible. Another possibility for bias is the accessibility and cost of participation. The participation fee was low and partially covered by the clinics in which the participants worked. Furthermore, representativeness in terms of occupational groups, gender, age and experience in Balint groups was surveyed. The aim was to avoid systematic distortions, such as participating in the group only at an early or late stage.

### Study size

There were 600 participants of Balint groups that were consecutively included in the study. This sample size should be sufficient to adequately assess the validity of the BGQ-C. Given the frequency of Balint groups in China, it should have taken 12 months to recruit this number. It was assumed in the design of the study that the distribution of the individual items would not permit the use of a confirmatory factor analysis with a maximum likelihood estimator. Therefore, the plan was to use estimators for categorically ordered data; however, this has a greater sample size requirement.

### Ethics approval

An informed consent document was used to explain the aims of the study to the participants and the leaders. The participants and leaders were informed that participation was voluntary, the data would be evaluated anonymously, and there would be no disadvantages if they chose not to participate in the study. By signing the document, the participants confirmed that they had been informed and agreed to the evaluation and processing of the collected data. The study was approved by the institutional review board of Peking Union Medical College Hospital in China and the institutional review board of the University of Freiburg in Germany.

### Translation procedure

The questionnaires were translated and back-translated into Mandarin Chinese based on a state-of-the-art translation procedure in accordance with the “ITC-Test Adaptation Guidelines” of the International Test Commission [28]. Requests for the Chinese version of the questionnaire can be addressed to the corresponding author.

### Statistical methods

Descriptive statistics were determined to test the BGQ-C (means, standard deviations, Cronbach’s alpha, and retest reliabilities). Since there were ceiling effects for several items, models for categorically ordered data were used for the structural equation models (WLSMV estimator). The discriminant validity was also checked against the group questionnaire [24]. The three-factor structure found in the German version (2017) [20] was tested within the Chinese and German-speaking samples. The fit of the models was rated by the suggestions of Schermelleh-Engel et al. [29]. In particular, the standardized root mean square residual (SRMR) should be below .10; the root mean square error of approximation (RMSEA) should be smaller than .05; and the Tucker-Lewis index (TLI) and the comparative fit index (CFI) should both be > .95. A multigroup comparison was performed to compare the factor loadings between German and Chinese participants. R 4.0.4, SPSS 27.0 and MPlus 8.5 were used.

## Results

### Characteristics of the participants in the Balint groups

One of the 831 questionnaires of the participants had to be eliminated due to implausible data, resulting in a sample of 830 questionnaires that could be evaluated. After excluding all participants with more than 5% missing values, the final sample size consisted of 806 participants from 123 groups led by 55 different leaders. Furthermore, the analysis of the missing values revealed a total of 25 missing values on items of the BGQ-C, which were unsystematically distributed among 22 participants (missing completely at random).

The sample of group participants (N = 806) consisted of 91% Han Chinese and 9% from other population groups. Sixty-two percent of the participants stated that their participation was voluntary and 35% that it was mandatory; 1% affirmed both statements. In the German-language comparison sample, approximately half of the participants were obligated to participate (51% of 1608). The Balint group experience of the Chinese participants was measured by the number of sessions they had attended. The median value of this variable was 2, with only 8 participants having attended 100 or more sessions. The range for this variable indicated that some completed the questionnaire after their first Balint group session.

There were 447 (55%) participants who were medical doctors. Of these, 240 named their specialty: general practitioners (4% of 447 doctors) represented a relatively small proportion compared to cardiologists (7%), with psychiatrists and psychologists being just over 10%. The remaining 143 (32%) physicians were spread across 35 other specialties. This was different from the distribution of professions among German-speaking participants, where 48% [20] of 1621 participants were general practitioners. Another difference was seen in the proportion of nurses who participated in Balint groups in China. Nineteen percent of the sample were nurses, whereas this professional group was not explicitly listed in the German-speaking sample (see Table 1).

**Table 1:** Gender, occupation, age and experience of participants and group leaders.

Characteristics		Participants, N = 806			Leaders, N = 55		
		N	%		N	%	
Gender	Male	205	25.4		20	36.3	
	Female	601	74.6		35	63.6	
Occupation	Psychiatrist	48	10.7^a^		30	54.5	
	Cardiologists	32	7.2^a^				
	General practitioners	16	3.6^a^		17	30.9	
	Gynecologist	15	3.4^a^				
	Internist	13	2.9^a^				
	Neurologist	12	2.7^a^				
	Anesthetist	11	2.5^a^				
	Emergency physician	11	2.5^a^				
	Radiologist	10	2.2^a^				
	Other specialties	71	15.8^a^				
	Nurses	157	19.5		2	3.6	
	Psychologists/Psychotherapists	65	8.1		6^b^	10.9	
	Students	81	10				
	Pedagogues	22	2.7		-		
	Others	44	5.5				
		**N**	**M (SD)**	**Median (Range)**	**N**	**M (SD)**	**Median (Range)**
	Age (years)	675	34.21 (9.44)	31 (19-64)	55	40.89 (5.20)	40 (32-48)
	Professional experience (years)	764	9.42 (9.58)	5 (0-40)	55	16.02 (7.10)	16 (2-28)
	Balint experience	792	7.86^c^ (31.82)	2^c^ (0-750)^c^	55	4.93^d^ (3.07)^d^	4^d^ (0-12)^d^

^a^ Percent of 447 doctors, of whom 240 indicated their specialty. ^b^ Three of the leaders indicated two specialties; they were only counted in the others category. Mean (M); standard deviation (SD). ^c^ Balint experience expressed as number of attended sessions. ^d^ Balint experience expressed in years.

The 55 Chinese leaders lead between 1 and 9 Balint groups. The Balint experience was measured by the number of group sessions conducted to date. Sixty-nine percent (38 of 55) of the leaders had led fewer than 100 Balint group sessions; 27% had led between 100 and 500 sessions. The German-speaking leaders had an average of 16.5 years of experience as a Balint group leader and 33 years of professional experience, which corresponds to twice and four times the experience of the Chinese leaders (see Table 1). The range of professional experience for the German-speaking leaders was between 8-59 years and the range for Balint group experience was between 1-45 years. In contrast, the Chinese leaders stated a maximum of 28 years of professional experience and 12 years of Balint experience. The professions of the leaders are listed in Table 1.

### Item distribution

The frequency distributions of all BGQ-C items showed ceiling effects (see Table 2). All item distributions were unimodal. The modal values were 4. The mean values show a high agreement of the participants on average. The present ceiling effects limit the differentiation ability of the items, although the item selectivities were still within the acceptable range [30]. The item analysis showed that the data do not follow the normal distribution; therefore, methods for ordinally scaled variables with ordered categories had to be used for inferential statistical analyses. The frequency distributions of the German-speaking sample also showed ceiling effects, but these were less pronounced than in the Chinese sample.

**Table 2:** BGQ-C item characteristics.

Item	N	M	Median	SD	Skew	Kurtosis	r_it_	p_m_
Scale 1: Reflection of transference dynamics in the doctor-patient relationship								
2	804	3.96	4	1.08	-1.12	1.1	0.66	79.2
10	804	3.84	4	1.05	-0.92	0.76	0.76	76.8
13	806	3.78	4	1.11	-1.08	1.13	0.58	75.6
15	806	3.65	4	1.06	-0.73	0.4	0.78	73
16	806	3.86	4	1	-0.79	0.54	0.75	77.2
Scale 2: Emotional and cognitive learning								
5	804	3.41	4	1.26	-0.62	-0.26	0.71	68.2
6	804	3.64	4	1.18	-0.74	0.01	0.72	72.8
9	804	4	4	1.02	-1.11	1.1	0.77	80
11	806	3.79	4	1.06	-0.78	0.24	0.8	75.8
Scale 3: Case mirroring in the dynamic of the group								
4	806	3.75	4	1.05	-0.92	0.94	0.61	75
7	802	3.83	4	1.07	-0.84	0.39	0.77	76.6
12	806	3.72	4	1.18	-0.88	0.38	0.55	74.4
Items without a scale								
1	802	3.62	4	1.15	-0.64	-0.18	0.63	72.4
3	803	3.9	4	0.99	-0.84	0.56	0.66	78
8	803	3.98	4	1.03	-1.02	0.83	0.58	79.6
14	805	3.46	4	1.2	-0.61	0	0.75	69.2
17	806	3.39	4	1.34	-0.68	-0.19	0.6	67.8

### Factor analyses

Since the questionnaire response format was in ordered categories and the frequency distributions showed clear ceiling effects, the WLSMV (Weighted Least Squares with Means and Variances adjusted) estimator was used for the following analyses. A total of 806 subjects were included in the confirmatory factor analysis. Since all factor loadings with values between .63 and .87 (see Table 3) became significant, the local fit can be considered acceptable. Despite the significant χ2 value, the 3-factor model fits acceptably on the Chinese data, χ2 (51; N = 806) = 350.867, p < .001. The RMSEA of .085 (0.077; 0.094) is above the limit of .05 and argues against an acceptable global fit. The incremental fit indices CFI = .977 and TLI = .971 are not sample size dependent and are considered good. Similarly, the SRMR of .025 is important for a good global fit. Since the confirmatory factor analysis in the German sample [23] did not consider the ordinal scale level of the variables, the analysis was repeated for categorically ordered data. A total of 1596 subjects were included in the analysis of the German sample. Again, the influence of sample size on the fit indices must be considered when evaluating the results. The correlations in Table 4 between the three factors were higher in the Chinese sample than in the German sample. Compared to the results in the German sample [23], the correlations between the factors in the German-speaking sample were somewhat higher when the scale level is taken into account.

**Table 3:** Factor loadings for the three-factor model.

	Factor loadings	S.E.	Est./S.E.
Scale 1 Reflection			
Item 2	0.721^***^	0.018	39.132
Item 10	0.840^***^	0.012	68.897
Item 13	0.639^***^	0.021	29.831
Item 15	0.832^***^	0.013	63.907
Item 16	0.828^***^	0.013	61.484
Scale 2 Learning			
Item 5	0.757^***^	0.015	48.989
Item 6	0.788^***^	0.015	53.308
Item 9	0.853^***^	0.013	68.192
Item 11	0.877^***^	0.01	90.131
Scale 3 Group dynamic			
Item 4	0.659^***^	0.02	32.402
Item 7	0.846^***^	0.013	63.546
Item 12	0.639^***^	0.023	28.14

^***^p<.001. Standard error (S.E.).

**Table 4:** Correlations between the scales of the BGQ in China and Germany.

		Scale 1	Scale 2	Scale 3
Scale 1	China	1	0.909	0.902
Reflection of the transference dynamics	Germany	1	0.784	0.694
Scale 2	China		1	0.921
Emotional and cognitive learning	Germany		1	0.565
Scale 3	China			1
Case mirroring in the dynamics of the group	Germany			1

All correlations are significant, p < .001

### Reliability

Scale 1, "reflection of transference dynamics in the doctor-patient relationship", had a very good value regarding internal consistency, Cronbach's α = .85 (N = 802) (see Table 4). All correlations of the individual items with the corrected overall scale were at least r = .55 and can therefore be considered acceptable. The Spearman correlation of the scale calculated for test-retest reliability was r_s_ = .697, p < .01 (N = 43). The internal consistency of scale 2, "emotional and cognitive learning", resulted in a Cronbach's α = .86 (N = 800). The correlations of the individual items with the scale were all above r = .71. The analysis of the test-retest reliability for this scale showed a correlation of r_s_ = .680, p <.001. For scale 3, "case mirroring in the dynamics of the group", the value of internal consistency was Cronbach's α = .70 (N = 802), which was lower than the value of the other two scales. The correlations of the items to the scale were at least r = .52 above the threshold value [31] and can be considered acceptable. The reliability estimates from the test-retest analysis resulted in a significant Spearman correlation of the scale values at both measurement points, r_s_ = .430, p = .004. None of the items improved Cronbach's α when it was excluded from the scale.

### Validity

All correlations between the scales of the BGQ-C and the scales and subscales of the group questionnaire (GQ-C) showed significance (see Table 5). The common variance between the convergent scales was between 22% and 33%. There were weak negative correlations between the "negative relationship" scale and the BGQ-C scales, but they were also significant. The coefficients of determination were between R_S_^2^ = .014 and .044. These results indicated that both questionnaires partly captured similar latent constructs in the area of positive working relationships and group attachment. However, the BGQ-C also measured a construct other than the therapeutic relationship within the groups.

**Table 5:** Reliability estimates for the BGQ-C and Spearman correlations between the BGQ-C and group questionnaire scores.

		Reflection	Learning	Group dynamic	N
Internal consistency (Cronbach’s alpha)	α (N)	.85 (802)	.86 (800)	.70 (802)	
Retest reliability (Spearman corrected)	r_s_	.70^**^	.68^**^	.43^*^	43
Solidarity	Total	.553^***^	.574^***^	.531^***^	806
	Leader	.488^***^	.506^***^	.465^***^	806
	Member	.506^***^	.512^***^	.493^***^	806
	Group	.533^***^	.554^***^	.501^***^	806
Positive working relationship	Total	.521^***^	.557^***^	.503^***^	806
	Leader	.505^***^	.551^***^	.482^***^	806
	Member	.496^***^	.521^***^	.484^***^	806
Negative relationship	Total	-.151^***^	-.209^***^	-.152^***^	806
	Leader	-.126^***^	-.166^***^	-.117^***^	806
	Member	-.149^***^	-.200^***^	-.144^***^	806
	Group	-.130^***^	-.175^***^	-.132^***^	806

^*^p <.05, ^**^ p <.01, ^***^ p<.001

### Multigroup comparison

An equation of the path weights between the German-speaking and the Chinese sample on the three-factor model based on Flatten et al. [20] showed significant differences in the path weights. The χ2 difference test, χ2(9) = 33.453; p = .0001, showed a significant reduction in model fit by equating the nonstandardized path weights. Thus, different compositions of the constructs in the two samples must be assumed.

## Discussion

### The 5 dimensions of Balint work

The focus of the Balint work is the doctor-patient relationship. For Balint, the doctor's personality is like medicine, with effects and side effects [4]. Information about the doctor-patient relationship is expressed in the dynamic interactions between doctors and patients, as well as in the phenomena of transference and countertransference.

With the BGQ in the study presented here, a new questionnaire instrument was developed in Germany and validated in China. For the first time, this instrument enabled the perceptions and impressions of participants in Balint group sessions to be recorded immediately after a session. It enabled the differentiation of group-dynamic and person-related dimensions. The 3 empirically identified scales resulted in a good model fit with the theoretical dimensions of the Balint work postulated in the literature. The test statistical parameters on the scales can be described as satisfactory to good.

Scale 1 (reflection of transference dynamics in the doctor-patient relationship) comprises the two theoretically postulated dimensions "diagnostics of the doctor-patient relationship" (transfer dynamics) and "creating awareness of one’s own proportionate contributions to the doctor-patient relationship". Scale 2 (emotional and cognitive learning) corresponds to the dimension "learning experience of medical participants with regard to the doctor-patient relationship". Scale 3 (case mirroring in the dynamic of the group) covers the dimension "reflection of the presented patient case in the group processes".

The BGQ thus covers the central content of 4 of the 5 postulated theoretical dimensions of Balint work. No independent dimension was found for the meaning or influence of the group leader (“significance of group leader interventions”) that has been considered in theoretical models.

### Significance of the Balint group leader

As group therapy research shows, the personality of the group leader is an important variable for the group process [32]. Surprisingly, all items designed to test the influence of interventions by the Balint group leader were eliminated during the 2 pilot studies in Germany, and therefore, the 17-item questionnaire does not cover and cannot confirm the hypothesized theoretical dimension “significance of group leader interventions”. This might be due to insufficient phrasing of the initial items, or it may reflect the subjective impressions of the doctors participating in the pilot studies that there was no influence by the group leader.

Nevertheless, our personal opinion regarding this aspect is that group leader interventions have an important impact on group processes. More research in this regard is needed.

### Validity and reliability of the Chinese BGQ

An acceptable fit of the three-factor model was shown with the Chinese dataset. Thus, this result basically supported the validity of the original model. However, the high correlations between the factors in the Chinese sample suggested the question of whether there may not be a general factor in the Chinese population after all. However, due to the lack of a theoretical foundation and better comparisons with data from other countries, we still recommend the use of the original model if it fits. It could be that group participants were better able to differentiate between the various constructs within the questionnaire after more sessions.

The correlations with the GQ-C scales speak for the construct validity of the BGQ-C. The BGQ-C scales seem to partly reflect the same aspects as the GQ-C, but there is still much variance that was not common. In particular, the low correlations of the BGQ-C with the scale “negative relationship” speak for a delimitation of the recorded constructs. The BGQ-C scales reflect conflicts and lack of empathy only to a small extent, although it can be assumed that conflicts between the participants of a Balint group have negative influences on the learning processes of the group.

The reliability of the Chinese version of the BGQ-C can be considered good. For scales 1 and 2, the internal consistencies were very good, and for scale 3, they were good. In addition, the comprehensibility of items 3 and 14 (no scale) and items 6 and 9 (scale 2) may be doubtful since several statements were linked. Another research question would be to develop a separate factor structure for China. However, this is beyond the scope of this paper.

### Representativeness

Most of the participants (55%) were doctors. Almost all clinical specialties were represented. The second largest occupational group was nurses (19.5%). This indicated that the two professional groups that provide immediate patient care were represented. The high proportion of nurses who are interested in Balint's work is encouraging. The composition of the participants is typical for Balint groups in China [18]. It represents the two important professional groups in general hospitals and important target groups for Balint's work. We are not aware of any larger, multicenter studies that could serve as a comparison.

We used a random technique to select our sample that will be highly representative.

The age and gender of the sample are representative of the two major professional groups, namely, doctors and nurses.

Psychiatrists and psychologists (65.4%) were represented much more frequently among the group leaders than among the participants (18.8%). Additionally, internationally, most group leaders came from the mental health field.

Most participants came from Beijing, Guangzhou and Shanghai. They represent major cities in North, East and South China. Rural regions were hardly represented.

The questionnaire was completed by the participants immediately after the session ended. This kept the nonresponse rate very low and increased generalization.

### Limitations

The item analysis showed ceiling effects for all items in the Chinese sample. These ceiling effects indicate that the items were too easily agreed upon, which limits the questionnaire's ability to differentiate. This effect is related to various phenomena. First, it indicates a yes-saying tendency of the participants. To counteract this tendency, it would be useful to formulate some questions in negative terms. This would further allow an assessment of whether the participants understood the content of the questions. Second, the ceiling effect could also be influenced by the fact that the participants answered in a very socially desirable way. The collection of social desirability data could provide information about how strong this confound is. Third, the ceiling effect can be explained in connection with the high correlations between the factors with a global positive judgment of the meeting. Previous research has shown that Balint groups are perceived as "good" by participants [33]. It is likely that cultural differences in the teaching style and in the public handling of differences in opinion also had an influence here.

This study was a cross-sectional design, and therefore, it is impossible to infer causality.

### Future steps

As the next steps, we will examine the following questions: Is there a difference in the learning effects between somatically oriented doctors and psychiatrists? Since nearly half of the participants considered participation mandatory: Is there a difference between obliged versus facultative participation? What influence does the presentation of a case have compared to participation without a case presentation and what influence does the group leader have on the learning effects?

Because of the high correlations between the factors in the Chinese sample, we suggest a country-specific evaluation of the data for intercultural studies. The item construction should also be revised to further improve the quality of the instrument. In this context, a qualitative study that examines the understanding of the items in more detail would also be useful. To enable further international comparative studies on Balint group work, ideally after a revision of the original, both an English version and other translations should be validated to examine further possible uses of the BGQ.

A short scale with 12 items representing the three relevant dimensions of Balint group work could be developed in a revision of the BGQ-C.

## Conclusions

In summary, the presented BGQ-C is a quick-to-complete measuring instrument based on items that allow the relevant dimensions of Balint group work to be recorded. These are “reflection of transference dynamics in the doctor-patient relationship”, “emotional and cognitive learning” and “case mirroring in the dynamic of the group”. The resulting three-factor solution can be assigned to 4 out of the abovementioned 5 dimensions of Balint’s theory of group work with physicians.

The BGQ-C can be used in Balint group daily clinical practice and for research. It allows the practitioner to use it as rapid feedback on the perceptions of participants in past group sessions and their learning experiences, which can be followed over the course of several sessions and thereby guide future work. The focus on affective perceptions of the presented treatment case and the freest possible development of an interaction between group members induced by presentation of the case are among the fundamental principles of Balint work. This process becomes transparent with the aid of the BGQ-C, and thus, the targeted improvement of the doctor-patient relationship can be examined. The BGQ is now available in German, English, Russian and Chinese versions.

## Data Availability

The datasets used and analyzed for the current study are available from the corresponding author on reasonable request.

## Abbreviations

BGQ-C: Mandarin version of the Balint group session questionnaire
BGQ-G: German version of the Balint group session questionnaire
GQ: Group questionnaire
GQ-C: Mandarin version of the group questionnaire
GQ-G: German version of the group questionnaire
DGB: German Balint Society
WLSMV: Weighted least squares with means and variances adjusted
SRMR: Standardized root mean square residual
RMSEA: Root mean square error of approximation
TLI: Tucker-Lewis index
CFI: Comparative fit index

## References

[1] Balint M, Balint E (1961). Psychotherapeutic techniques in medicine.

[2] Rabin S, Maoz B, Elata-Alster G (1999). Doctors' narratives in Balint groups. Br J Med Psychol..

[3] Rabin S, Maoz B, Shorer Y, Matalon A (2009). Balint groups as 'shared care' in the area of mental health in primary medicine. Ment Health Fam Med..

[4] Balint M (1957). The doctor, his patient and the illness.

[5] Van Roy K, Vanheule S, Inslegers R (2015). Research on Balint groups: a literature review. Patient Educ Couns..

[6] Kutter P, Körner J, Neubig H, Rosin U (1990). Empathy training in Balint groups: research on psychology students. Balint group in clinic and practice.

[7] Cataldo KP, Peeden K, Geesey ME, Dickerson L (2005). Association between Balint training and physician empathy and work satisfaction. Fam Med..

[8] Obliers R, Köhle K, Kaerger H, Bahrs O (1996). Video-documentation as a tool to insure quality assurance: evaluation of the development of medical conversational behaviour after attending a Balint group. Contributions to medical psychology and medical sociology.

[9] Benson J, Magraith K (2005). Compassion fatigue and burnout: the role of Balint groups. Aust Fam Physician..

[10] Kjeldmand D, Holmström I (2008). Balint groups as a means to increase job satisfaction and prevent burnout among general practitioners. Ann Fam Med..

[11] Ghetti C, Chang J, Gosman G (2009). Burnout, psychological skills, and empathy: balint training in obstetrics and gynecology residents. J Grad Med Educ..

[12] Bar-Sela G, Lulav-Grinwald D, Mitnik I (2012). "Balint group" meetings for oncology residents as a tool to improve therapeutic communication skills and reduce burnout level. J Cancer Educ..

[13] Popa-Velea O, Trutescu CI, Diaconescu LV (2019). The impact of Balint work on alexithymia, perceived stress, perceived social support and burnout among physicians working in palliative care: a longitudinal study. Int J Occup Med Environ Health..

[14] Editorial. Chinese doctors are under threat. Lancet. 2010;376:657. 10.1016/S0140-6736(10)61315-3 20801385

[15] Editorial. Ending violence against doctors in China. Lancet. 2012;379:1764. 10.1016/S0140-6736(12)60729-6 22579308

[16] Jie L (2012). New generations of Chinese doctors face crisis. Lancet..

[17] Fritzsche K, Scheib P, Ko N, Wirsching M, Kuhnert A, Hick J (2012). Results of a psychosomatic training program in China, Vietnam and Laos: successful cross-cultural transfer of a postgraduate training program for medical doctors. BioPsychoSoc Med..

[18] Jing W, Otten H, Sullivan L, Lovell-Simons L, Granek-Catarivas M, Fritzsche K (2013). Improving the doctor-patient relationship in China: the role of balint groups. Int J Psychiatry Med..

[19] Cao J, Shi L, Zhao X, Hong X, Xiong N, Wie J (2014). Study on current needs and future development possibilities of professional doctor-patient relationship skill (Balint group work) in China. (In Chinese) Chin J Med Edu..

[20] Lu D, Lan M, Zhang N (2020). Intervention of Balint group on the emotional labor and job burnout of nurses in cardiology. Zhonghua Lao Dong Wei Sheng Zhi Ye Bing Za Zhi..

[21] Huang L, Harsh J, Cui H, Wu J, Thai J, Zhang X (2019). A Randomized controlled trial of Balint groups to prevent burnout among residents in China. Front Psychiatry..

[22] Yang C, Zhou B, Wang J, Pan S (2021). The effect of a short-term Balint group on the communication ability and self-efficacy of pre-examination and triage nurses during COVID-19. J Clin Nurs..

[23] Flatten G, Tschuschke V. How beneficial are Balint groups? In: Proceedings of the 20th International Balint Congress. 2017. p. 150-70.

[24] Krogel J (2009). The group questionnaire: a new measure of the group relationship.

[25] Balint M (1969). The structure of the training-cum-research-seminars. its implications for medicine. J R Coll Gen Pract..

[26] Johnson J, Burlingame G, Olsen J, Davies D, Gleave R (2005). Group climate, cohesion, alliance, and empathy in group psychotherapy: multilevel structural equation models. J Couns Psychol..

[27] Bormann B, Burlingame GM, Strauß B (2011). The group questionnaire (GQ-D). Instrument to measure therapeutic relationships in group psychotherapy. Psychotherapeut..

[28] Hambleton RK, Merenda PF, Spielberger CD (2005). Adapting educational and psychological tests for cross-cultural assessment.

[29] Schermelleh-Engel K, Moosbrugger H, Müller H (2003). Evaluating the fit of structural equation models: tests of significance and descriptive goodness-of-fit measures. Methods Psychol Res Online..

[30] Moosbrugger H, Kelava A. Test theory and construction of questionnaires. (in German) Heidelberg: Springer; 2012.

[31] Field A (2013). Discovering statistics using IBM SPSS statistics.

[32] Tschuschke V, Greene LR (2002). Group therapists' training: what predicts learning?. Int J Group Psychother..

[33] Rosin U, Körner J, Neubig H, Rosin U (1989). Balint groups: design – research – results. The balint group in clinic and practice.

